# Alcohol-positive polytrauma patients: injury patterns, ICU resource use and mortality at a German Level I trauma center

**DOI:** 10.1186/s12245-026-01411-x

**Published:** 2026-10-20

**Authors:** Claudius Thiedemann, Sergei Gorovoi, Lorenz Huber, Daniel Popp, Michael Galler, Paul Schmitz, Volker Alt, Antonio Ernstberger

**Affiliations:** 1 Department of Trauma Surgery, University Hospital Regensburg, Franz-Josef-Strauss-Allee 11, 93053 Regensburg, Germany; 2 Department of Trauma Surgery, Caritas Hospital St. Josef Regensburg, Regensburg, Germany

**Keywords:** Multiple trauma, Severely injured patient, Alcohol, Trauma bay, Emergency room, Intoxication, Polytrauma, Major trauma

## Abstract

**Background:**

In most countries, alcohol consumption is so culturally ingrained that even small amounts are permitted while driving. Even at a blood alcohol concentration of 0.5 g/L, ethanol impairs mental and cognitive performance. Studies from the US and Europe estimate that elevated blood alcohol levels may be detected in 10%–40% of all emergency department patients. Against this background, we investigated the impact of alcohol consumption on injury severity, injury patterns, as well as primary and secondary outcomes in polytrauma patients.

**Materials and Methods:**

This retrospective, single-center cohort study included patients admitted to a Level I trauma center via the trauma room and documented in the TraumaRegister DGU^®^ between 2015 and 2023. Serum ethanol was determined routinely in all adolescent and adult trauma room patients. Patients without serum ethanol measurement or younger than 13 years were excluded. Patients with detectable serum ethanol (> 1 mg/dL, detection limit of the laboratory; ALC) were compared with patients without (noALC) using the χ² and Mann–Whitney U tests. Multivariable logistic and linear regression analyses were adjusted for age, sex, Injury Severity Score (ISS), severe traumatic brain injury (TBI) and pre-injury ASA classification. A dose–response analysis was performed using serum ethanol categories.

**Results:**

A total of 1,524 patients were analyzed: 431 (28.3%) were alcohol-positive (ALC) and 1,093 (71.7%) were not (noALC). ALC patients were younger, more often male and had a lower prehospital Glasgow Coma Scale (GCS) score (*p* < 0.001), whereas the ISS did not differ (*p* = 0.396). They were more frequently victims of violent crime and were overrepresented among pedestrians and falls from < 3 m, whereas motorcyclists were more often sober. Alcohol-positive patients were more often treated in the ICU (90.0% vs. 86.3%; *p* = 0.048), but this association was attenuated after multivariable adjustment (adjusted odds ratio [aOR] 1.40; 95% CI 0.96–2.03). Length of ICU stay and duration of mechanical ventilation did not differ, and alcohol was not independently associated with mortality (aOR 0.91; 95% CI 0.56–1.47). Severe TBI was more frequent in alcohol-positive patients (38.5% vs. 29.5%), increased with serum ethanol concentration, and was the strongest predictor of ICU treatment and mortality.

**Conclusion:**

Nearly one in three severely injured patients had detectable serum ethanol. After adjustment for confounders, alcohol was not independently associated with mortality or intensive care resource use, and no dose–response relationship was found for these outcomes. The more frequent ICU treatment of alcohol-positive patients appears to be explained by their distinct injury pattern, particularly the concentration-dependent increase in severe TBI.

**Supplementary Information:**

The online version contains supplementary material available at https://doi.org/10.1186/s12245-026-01411-x.

## Background

Alcohol is a major risk factor for injury: even at low concentrations, it impairs motor skills, reaction time and coordination [1, 2], and there is no safe level of consumption [3]. Alcohol is involved in 40–56% of violent crimes and 30% of road traffic accidents [4, 5], and elevated blood alcohol concentrations are found in 10–40% of emergency department patients [4]. Europe is the region with the highest alcohol consumption worldwide [3].

In severely injured patients, however, the impact of alcohol on outcome remains controversial. While acute alcohol exposure is a risk factor for traumatic brain injury (TBI), several studies have reported an association with lower mortality after head injury [6–8]. Most available data derive from registry analyses in which blood alcohol is documented only when determined at the discretion of the treating team [8, 9], and data on in-hospital resource use, particularly intensive care, are scarce.

At our Level I trauma center, serum alcohol concentration is determined routinely in all adolescent and adult trauma room patients, which minimizes selection towards clinically apparent intoxication. Combining registry data with a detailed, prospectively collected institutional dataset, we aimed to answer the following questions:

1. What impact does documented alcohol consumption have on the primary endpoint of mortality in patients with multiple trauma?
2. What secondary endpoints emerge when comparing these patients to non-alcohol-impaired patients in terms of injury severity, injury patterns, hospital stay, and length of stay in the intensive care unit?
3. Is there a dose–response relationship between serum ethanol concentration and injury pattern or outcome?

## Methods

In a Level I trauma center, trauma room patients were analyzed in a retrospective cohort study over a 9-year period (2015–2023). The study is reported in accordance with the STROBE statement.

Data were collected using variables from the TraumaRegister DGU^®^ [10] as well as from a prospectively collected in-house dataset containing more than 350 variables on trauma room care. Data pertaining to the emergency department were prospectively collected by research assistants during the respective emergency department care. All data were checked for plausibility and verified against the original clinical and laboratory records where necessary.

Patient care in the trauma room was provided according to the established ATLS^®^ protocol, which was adapted to the hospital’s specific conditions. The study included trauma room patients who were admitted to the ICU with a MAIS score of ≥ 2 (Maximum Abbreviated Injury Scale), died in the trauma room, or had a MAIS score of ≥ 3 and were admitted to the general ward. Serum ethanol concentration was determined routinely in all adolescent and adult trauma room patients. Excluded were patients for whom the dataset contained no information on laboratory-measured serum ethanol (*n* = 172) or who were younger than 13 years of age (*n* = 107). Due to the increasingly early onset of alcohol consumption and the inclusion of younger age groups in statistical analyses [11, 12] as well as clinical experience, this study also deliberately included teenage patients and set this age limit. In addition to demographic data, the study evaluated patient care in the emergency room, radiological imaging, and the subsequent course of patients, including surgeries and intensive care monitoring through to discharge from the hospital. Furthermore, injury severity was calculated using the ISS (Injury Severity Score), the NISS (New Injury Severity Score), and the maximum AIS. Severe TBI was defined as an AIS head score ≥ 3; pre-injury health status was assessed using the American Society of Anesthesiologists (ASA) classification.

Patients with detectable serum ethanol (> 1 mg/dL [0.01 g/L], i.e., above the detection limit of the laboratory; ALC) were compared with patients without detectable serum ethanol (noALC). Serum ethanol is reported in mg/dL; for orientation, whole-blood equivalents (‰) were calculated using a serum-to-whole-blood ratio of 1.236. For the dose–response analysis, alcohol-positive patients were categorized according to the legally relevant whole-blood thresholds in Germany, corresponding to serum concentrations of < 62 mg/dL (< 0.5‰, limit for driving), 62–< 136 mg/dL (0.5–< 1.1‰; 1.1‰ = absolute unfitness to drive), 136–< 247 mg/dL (1.1–< 2.0‰) and ≥ 247 mg/dL (≥ 2.0‰, marked intoxication suggesting habituation).

As continuous variables were not normally distributed according to the Kolmogorov–Smirnov test, they are reported as median and interquartile range (IQR) and were compared using the Mann–Whitney U test. Categorical variables are reported as number and percentage and were compared using the χ² test. Trends across serum ethanol categories were assessed using the linear-by-linear association test and the Jonckheere–Terpstra test. Patients excluded because of missing serum ethanol were compared with the study population (Supplementary Table S1).

To account for confounding, multivariable logistic regression was performed for in-hospital mortality and ICU admission, and multivariable linear regression for the log-transformed length of ICU stay (ICU patients only) and duration of mechanical ventilation (ventilated patients only). Covariates were selected a priori: age, sex, ISS, severe TBI (AIS head ≥ 3) and pre-injury ASA classification (≥ 3 vs. 1–2). As alcohol itself lowers the GCS, the prehospital GCS was considered a potential mediator and included only in a sensitivity analysis. Results are reported as adjusted odds ratios (aOR) or relative change with 95% confidence intervals (CI). Missing ICU or ventilation days were coded as 0 (< 24 h); otherwise, a complete-case analysis was performed. Given the number of comparisons, analyses of secondary endpoints were considered exploratory. A p-value < 0.05 was considered statistically significant. Analyses were performed using IBM SPSS Statistics, version 31 (IBM Corp., Armonk, NY, USA).

The standardized mortality ratio (SMR) was calculated as the ratio of observed to expected deaths, with expected mortality based on the Revised Injury Severity Classification II (RISC II) [13]; 95% CIs were calculated assuming a Poisson distribution. SMR analyses were restricted to patients with available RISC II.

The study was approved by the Ethics Committee of the University of Regensburg (No. 26-4624-104). Owing to the retrospective analysis of pseudonymized routine data, no additional informed consent was required.

## Results

Over a 9-year period (2015–2023), a total of 1,803 patients were initially enrolled. After exclusion of 107 patients younger than 13 years and 172 patients without serum ethanol measurement, the study population comprised 1,524 patients (Supplementary Figure S1), of whom 431 (28.3%) were assigned to the ALC group and 1,093 (71.7%) to the noALC group (Table 1). Patients in the ALC group were younger (median 47 vs. 50 years; *p* = 0.005), more often male (81.7% vs. 72.2%; *p* < 0.001) and had a lower prehospital GCS (median 14 vs. 15; *p* < 0.001).

Compared with the study population, the 172 excluded patients had a higher in-hospital mortality (22.7% vs. 11.9%; *p* < 0.001). Of these, 22 (12.9%) died in the trauma room or within 24 h of admission (study population: 4.0%; *p* < 0.001); these patients had non-survivable injuries (median ISS 40.5, median prehospital GCS 3). The remaining 149 excluded patients did not differ from the study population with regard to age, pre-existing conditions, mortality or length of hospital stay (Supplementary Table S1).

### Demographics

**Table 1:** Demographic data and injury severity of the noALC and ALC groups

	noALC (*n* = 1,093)	ALC (*n* = 431)	*p*-value
Age, years	50 (28–66)	47 (28–59)	0.005
Male sex	789 (72.2)	352 (81.7)	< 0.001
ASA ≥ 3	130 (12.2)	38 (9.0)	0.075
ISS	17 (10–27)	17 (10–25)	0.396
NISS	22 (13–34)	22 (12–34)	0.596
RISC II predicted mortality, %	0.8 (0.3–5.2)	0.9 (0.3–5.2)	0.534
Serum ethanol, mg/dL	–	95 (16–209)	–
Whole-blood equivalent, ‰ *	–	0.77 (0.13–1.69)	–

* Calculated as serum ethanol / 1.236

Values are median (IQR) or n (%). ISS: Injury Severity Score; NISS: New Injury Severity Score; RISC II: Revised Injury Severity Classification II; ASA: American Society of Anesthesiologists classification

ISS and NISS did not differ between the groups. Among alcohol-positive patients, the median serum ethanol concentration was 95 mg/dL (IQR 16–209), corresponding to approximately 0.77‰ in whole blood (Table 1).

Rates of direct transfer from the trauma room to the operating room (30.6% vs. 34.6%; *p* = 0.140) or to the ICU (55.5% vs. 52.2%; *p* = 0.258) did not differ. Alcohol-positive patients were more often treated in the ICU during their hospital stay (90.0% vs. 86.3%; *p* = 0.048), whereas length of ICU stay (median 3.5 vs. 3 days; *p* = 0.725), duration of mechanical ventilation (*p* = 0.808) and total length of hospital stay (*p* = 0.651) were similar (Table 3).

### Prehospital

**Table 2:** Prehospital data, causes and mechanisms of injury of the noALC and ALC groups

	noALC (*n* = 1,093)	ALC (*n* = 431)	*p*-value
Cause: accident	1,032 (94.4)	398 (92.3)	0.129
Cause: suspected violent crime	13 (1.2)	12 (2.8)	0.027
Penetrating trauma	31 (2.9)	15 (3.5)	0.510
Motorcycle accident	205 (18.8)	47 (10.9)	< 0.001
Bicycle accident	98 (9.0)	50 (11.6)	0.118
Pedestrian	27 (2.5)	21 (4.9)	0.016
Fall < 3 m	171 (15.6)	101 (23.4)	< 0.001
Fall ≥ 3 m	120 (11.0)	52 (12.1)	0.546
Intubated on arrival	326 (33.2)	143 (36.9)	0.203
Prehospital GCS	15 (12–15)	14 (7–15)	< 0.001

Values are n (%) or median (IQR). GCS: Glasgow Coma Scale

Patients in the ALC group were more often victims of violent crime (2.8% vs. 1.2%; *p* = 0.027), whereas penetrating trauma was not more common (3.5% vs. 2.9%; *p* = 0.510). Motorcyclists were more often sober (10.9% of ALC vs. 18.8% of noALC patients; *p* < 0.001), whereas alcohol-positive patients were more frequently injured as pedestrians (4.9% vs. 2.5%; *p* = 0.016) and in falls from < 3 m (23.4% vs. 15.6%; *p* < 0.001). No differences were observed for cyclists or falls from ≥ 3 m. The proportion of patients intubated on arrival did not differ (36.9% vs. 33.2%; *p* = 0.203) (Table 2).

### Treatment/Outcome

**Table 3:** Trauma room care, injury pattern and outcome of the noALC and ALC groups

	noALC (*n* = 1,093)	ALC (*n* = 431)	*p*-value
Whole-body CT	923 (84.6)	365 (85.1)	0.815
AIS head	1 (0–3)	2 (0–3)	< 0.001
AIS face	0 (0–1)	0 (0–1)	0.009
AIS thorax	2 (0–3)	1 (0–3)	0.073
AIS abdomen	0 (0–0)	0 (0–0)	0.910
AIS spine	0 (0–2)	0 (0–2)	0.001
AIS upper extremities	0 (0–2)	0 (0–2)	0.064
AIS lower extremities	0 (0–2)	0 (0–1)	< 0.001
Severe TBI (AIS head ≥ 3)	322 (29.5)	166 (38.5)	< 0.001
Transfer trauma room → OR	378 (34.6)	132 (30.6)	0.140
Transfer trauma room → ICU	571 (52.2)	239 (55.5)	0.258
Number of surgical procedures	2 (0–4)	2 (0–4)	0.419
PRBC units within 48 h, mean (SD)	1.1 (4.3)	0.7 (2.4)	0.140
ICU treatment	943 (86.3)	388 (90.0)	0.048
Length of ICU stay, days *	3 (2–9)	3.5 (2–11)	0.725
Mechanical ventilation in ICU	471 (46.4)	206 (50.7)	0.139
Duration of ventilation, days †	3 (1–9)	3 (1–11)	0.808
Total length of hospital stay, days	12 (6–20)	12 (5–21)	0.651
In-hospital mortality	139 (12.7)	43 (10.0)	0.137
SMR (95% CI) ‡	1.16 (0.97–1.38)	0.94 (0.67–1.27)	–

* ICU patients only; † ventilated patients only; ‡ patients with available RISC II (noALC *n* = 961, ALC *n* = 372). PRBC: Mann–Whitney U test

Values are median (IQR) or n (%). AIS: Abbreviated Injury Scale; TBI: traumatic brain injury; OR: operating room; ICU: intensive care unit; PRBC: packed red blood cells; SMR: standardized mortality ratio (observed/expected deaths based on RISC II)

Whole-body CT was performed in 85.1% and 84.6% of patients, respectively (*p* = 0.815). Alcohol-positive patients more frequently sustained head (*p* < 0.001) and facial injuries (*p* = 0.009) and severe TBI (38.5% vs. 29.5%; *p* < 0.001), whereas sober patients had higher AIS scores for the spine (*p* = 0.001) and the lower extremities (*p* < 0.001). Thoracic, abdominal and upper-extremity injuries did not differ significantly (Table 3).

In-hospital mortality was 10.0% in the ALC group and 12.7% in the noALC group (*p* = 0.137). The SMR based on RISC II was 0.94 (95% CI 0.67–1.27) in the ALC group and 1.16 (95% CI 0.97–1.38) in the noALC group (Table 3).

### Multivariable analysis

After adjustment for age, sex, ISS, severe TBI and ASA classification, alcohol was not independently associated with in-hospital mortality (aOR 0.91; 95% CI 0.56–1.47), length of ICU stay (+ 9%; 95% CI − 2% to + 20%) or duration of mechanical ventilation (+ 9%; 95% CI − 6% to + 27%) (Table 4). The crude association with ICU admission (OR 1.47; 95% CI 1.02–2.12) was attenuated after adjustment (aOR 1.40; 95% CI 0.96–2.03). Severe TBI, which was more frequent in the ALC group (38.5% vs. 29.5%), was the strongest independent predictor of mortality (aOR 4.66; 95% CI 2.95–7.36) and ICU admission (aOR 1.91; 95% CI 1.24–2.94). Results were consistent in the sensitivity analysis including prehospital GCS.

**Table 4:** Association of alcohol (ALC vs. noALC) with outcomes in crude and multivariable models

Outcome	Crude	Model 1	Model 2	Model 3
In-hospital mortality, OR	0.79 (0.55–1.14)	0.97 (0.66–1.42)	0.91 (0.56–1.47)	0.69 (0.38–1.23)
ICU admission, OR	1.47 (1.02–2.12)	1.47 (1.02–2.13)	1.40 (0.96–2.03)	1.43 (0.96–2.13)
Length of ICU stay *, %	+ 5 (− 5 to + 17)	+ 7 (− 4 to + 18)	+ 9 (− 2 to + 20)	+ 6 (− 5 to + 17)
Duration of ventilation †, %	+ 7 (− 7 to + 25)	+ 10 (− 6 to + 28)	+ 9 (− 6 to + 27)	+ 6 (− 9 to + 24)

*n* = 1,487 for logistic models (crude to Model 2) and 1,314 for Model 3. * ICU patients only (*n* = 1,301); † ventilated patients only (*n* = 657). In Model 2, severe TBI was associated with mortality (aOR 4.66; 95% CI 2.95–7.36) and ICU admission (aOR 1.91; 95% CI 1.24–2.94)

Values are odds ratios (logistic regression) or relative change in % (linear regression of ln[days + 1]) with 95% CI. Model 1: adjusted for age and sex; Model 2 (main model): additionally adjusted for ISS, severe TBI (AIS head ≥ 3) and ASA ≥ 3; Model 3 (sensitivity analysis): Model 2 plus prehospital GCS

### Dose–response analysis

Among alcohol-positive patients, 187 (43%) had a serum ethanol concentration of < 62 mg/dL, 60 (14%) of 62–< 136 mg/dL, 108 (25%) of 136–< 247 mg/dL and 76 (18%) of ≥ 247 mg/dL. The proportion of severe TBI increased with serum ethanol concentration (from 29.5% in sober patients to 46.3% at 136–< 247 mg/dL; p for trend < 0.001), whereas no dose–response relationship was found for mortality (p for trend = 0.139), length of ICU stay (*p* = 0.751) or duration of ventilation (*p* = 0.831). After adjustment, no ethanol category was independently associated with mortality or ICU admission (Supplementary Table S2).

## Discussion

Nearly one in three patients in the study group had a positive blood alcohol level, which is consistent with the results of previous studies [14]. The patient population was predominantly male and consisted of younger patients. These figures are also comparable to the majority of previous polytrauma studies [15, 16].

Regarding the causes of accidents, involvement in violent crimes is particularly noteworthy, which is consistent with the increased incidence of penetrating injuries that Wagner et al. were able to demonstrate in their study [9], although we cannot confirm this with our data. Significant differences between the two study populations in road traffic were found only among pedestrians and motorcyclists, representing a significant development compared to the non-significant differences reported by Brockamp et al. [8]. These injuries fall into the category of blunt trauma, which was also shown to be significant in the study by Afshar et al. [17]. In contrast, our data showed no significant differences for falls from heights over 3 m. However, falls from low heights (< 3 m) are particularly striking here.

It should be noted that the increasing use of increasingly modern safety devices such as airbags is one of the factors contributing to ever-improving protection [14]. Furthermore, it has been demonstrated that alcohol consumption impairs the sense of balance and leads to an increased risk of falls due to impaired protective reflexes [8].

Although ISS did not differ between the groups, the ALC group showed a significantly lower prehospital GCS, comparable to the study by Scheyerer et al. [18]. The lower prehospital GCS may partly explain why alcohol-positive patients were more often treated in the ICU, although length of ICU stay, duration of ventilation, direct transfer to the ICU, the number of surgical procedures and total length of hospital stay did not differ. However, after adjustment for injury severity, severe TBI and pre-injury health status, alcohol was no longer independently associated with ICU admission, length of ICU stay or ventilation duration. The more frequent intensive care treatment of alcohol-positive patients therefore seems to reflect their distinct injury pattern, in particular the higher rate of severe TBI, which increased with serum ethanol concentration, rather than an effect of alcohol intoxication itself. Since alcohol itself lowers the GCS, a reduced GCS in these patients does not necessarily reflect the severity of brain injury but may lead to more cautious monitoring. Thus, it can be postulated that demonstrable alcohol consumption neither protects patients from severe injuries nor shortens the duration of the total hospital stay, as Hsieh et al. have already demonstrated [19]. It should be noted, however, that the study by Stübig et al. demonstrated increased prehospital mortality among intoxicated patients [20] and even when significant correlations are found, alcohol consumption should not, of course, be regarded as the sole causal factor in all cases.

As Leonard et al. have already demonstrated in their review, alcohol consumption is associated with a slight increase in the incidence of complications in the hospital (sepsis and multiple organ failure) [21].

Wagner et al. reported findings contrary to our data, as they were unable to demonstrate significant differences in the GCS. However, they noted that alcohol consumption in trauma patients can be an important factor in acidosis, though without affecting the patient’s treatment outcome [9]. Regarding overall complications, Friedman et al. also demonstrated that cardiac complications decreased by over 23% and renal complications by 30% in intoxicated patients, while the risk of aspiration pneumonia and pancreatitis increased [22].

Mortality did not differ significantly between the groups (10.0% vs. 12.7%; *p* = 0.137), and the RISC II-based SMR was also comparable; this finding persisted after multivariable adjustment. Previous studies have reported lower mortality in alcohol-positive patients with severe TBI [8, 19], with higher alcohol concentrations being associated with better survival [6, 23]. Proposed mechanisms include reduced systemic IL-6 levels and immunomodulatory effects of alcohol [7]. As these mechanisms were not investigated in the present study, our data can neither confirm nor refute a neuroprotective effect of alcohol, and this hypothesis should be addressed in dedicated prospective studies. The higher rate of head and facial injuries in alcohol-positive patients is consistent with previous reports [19, 23] and may be explained by differences in injury mechanisms, such as low falls, pedestrian accidents and violence.

A strength of this study is the routine determination of serum alcohol in all adolescent and adult trauma room patients. In contrast to registry-based analyses, in which alcohol is documented only when measured at the clinician’s discretion [8, 9], this approach reflects the prevalence of alcohol exposure in an unselected cohort of severely injured patients. In addition, registry data were complemented by a prospectively collected institutional dataset of more than 350 variables on trauma room care, allowing a detailed characterization of initial management alongside the in-hospital course, including intensive care.

### Limitations

This study has several limitations. First, its retrospective single-center design limits generalizability, although treatment followed ATLS^®^ principles and the German S3 guideline on polytrauma care, comparable to other Level I trauma centers. Second, as an observational study, it can only demonstrate associations, not causal relationships. Although the main outcomes were adjusted for age, sex, injury severity, severe TBI and pre-injury health status, residual confounding cannot be excluded; although pre-injury health status was accounted for using the ASA classification, specific comorbidities and chronic alcohol use could not be assessed. Third, 172 patients (10.1%) without serum ethanol measurement were excluded (Supplementary Table S1). Of these, 22 (12.9%) died in the trauma room or within 24 h of admission. These patients had non-survivable injuries (median ISS 40.5, median prehospital GCS 3), and in most cases, a decision to withdraw treatment was made early, so that no laboratory tests were obtained. The remaining 149 excluded patients did not differ from the study population with regard to age, pre-existing conditions, mortality or length of hospital stay. A relevant selection bias therefore appears unlikely; it mainly concerns patients with non-survivable injuries, whose outcome would not have been influenced by alcohol. Fourth, given the number of comparisons, analyses of secondary endpoints should be considered exploratory, and type I errors cannot be ruled out. Finally, other psychoactive substances were not systematically assessed. In view of the recent legalization of cannabis in Germany, future studies should also address the impact of other psychoactive substances on severely injured patients.

## Conclusion

In this single-center cohort, nearly one in three severely injured patients had a detectable serum alcohol concentration. Alcohol-positive patients differed from sober patients in their injury pattern, with more severe TBI, facial injuries, violence-related injuries and low falls. After adjustment for relevant confounders, however, alcohol was not independently associated with mortality, ICU admission, length of ICU stay or duration of mechanical ventilation. The more frequent intensive care treatment of alcohol-positive patients thus appears to reflect their injury pattern rather than an independent effect of alcohol. A causal relationship cannot be inferred from the present data.

## Supplementary Information

Below is the link to the electronic supplementary material.

Supplementary Material 1: Additional file 1 (PDF): Supplementary Tables S1–S2 and Supplementary Figure S1. Table S1: Comparison of patients excluded because of missing serum ethanol with the study population. Table S2: Injury pattern and outcome according to serum ethanol category. Figure S1: Flow diagram of patient selection.

## Data Availability

The dataset generated and analyzed during the current study is not publicly accessible but is available from the corresponding author upon reasonable request.

## Abbreviations

DGU: Deutsche Gesellschaft für Unfallchirurgie (German Society for Trauma Surgery)
GCS: Glasgow Coma Scale
TBI: Traumatic Brain Injury
ÜTZ: Überregionales Trauma Zentrum (Level I trauma center)
ATLS: Advanced Trauma Life Support
ICU: Intensive Care Unit
MAIS: Maximum Abbreviated Injury Scale
ISS: Injury Severity Score
NISS: New Injury Severity Score
AIS: Abbreviated Injury Scale
RISC II: Revised Injury Severity Classification II
SD: Standard Deviation
CT: Computed Tomography
SMR: Standardized Mortality Ratio
aOR: adjusted odds ratio
ASA: American Society of Anesthesiologists
CI: Confidence interval
IQR: Interquartile range
PRBC: Packed red blood cells
STROBE: Strengthening the Reporting of Observational Studies in Epidemiology
