# Supplementary material for: Alcohol-positive polytrauma patients: injury patterns, ICU resource use and mortality at a German Level I trauma center
Source: Int J Emerg Med. 2026 Oct 20;19:269. doi: 10.1186/s12245-026-01411-x (PMC13682127; doi:10.1186/s12245-026-01411-x)

Additional file 1

Alcohol-positive polytrauma patients: injury patterns, ICU resource use and mortality at a German Level I trauma center

Supplementary Tables S1–S2 and Supplementary Figure S1

Supplementary Table S1: Comparison of patients excluded because of missing serum ethanol with the study population. Values are median (IQR) or n (%). p-values refer to the comparison with the study population (Mann–Whitney U test or χ² test).

	Study population (n = 1,524)	All excluded (n = 172)	p	Early deaths * (n = 22)	Other excluded (n = 149)	p
Age, years	49 (28–63)	53.5 (30–73)	0.051	56.5 (48–81)	52 (28–70)	0.275
Male sex	1,141 (74.9)	104 (60.5)	< 0.001	14 (63.6)	90 (60.4)	< 0.001
ASA ≥ 3	168 (11.3)	29 (17.6)	0.018	7	22 (14.9)	0.197
ISS	17 (10–27)	16 (9–26)	0.134	40.5 (25–75)	13 (9–24)	0.001
NISS	22 (13–34)	22 (11–34)	0.251	54.5 (26–75)	17 (10–29)	0.005
RISC II predicted mortality, %	0.8 (0.3–5.2)	1.3 (0.3–21.8)	0.044	97.8 (92.6–98.8)	0.8 (0.3–5.6)	0.345
Prehospital GCS	15 (11–15)	14 (8–15)	0.372	3 (3–4.5)	15 (13–15)	0.054
Suspected violent crime	25 (1.7)	2 (1.2)	0.629	0	2 (1.3)	0.776
Penetrating trauma	46 (3.1)	9 (5.4)	0.106	1	8 (5.5)	0.117
ICU treatment	1,331 (87.3)	126 (73.3)	< 0.001	4	122 (81.9)	0.060
Mechanical ventilation in ICU	677 (47.6)	60 (42.6)	0.248	4	56 (41.8)	0.195
Total length of hospital stay, days	12 (5–20)	8 (3–17)	< 0.001	1 (1–1)	10 (5–18)	0.216
Death in trauma room or ≤ 24 h	61 (4.0)	22 (12.9)	< 0.001	22 (100)	0	–
In-hospital mortality	182 (11.9)	39 (22.7)	< 0.001	22 (100)	16 (10.7)	0.664

* Died in the trauma room or within 24 hours of admission. One deceased excluded patient without documented time of death could not be assigned. ASA: American Society of Anesthesiologists classification.

Supplementary Table S2: Injury pattern and outcome according to serum ethanol category. Values are % or adjusted odds ratio (aOR, 95% CI; Model 2, reference: noALC). Whole-blood equivalents in parentheses.

	**noALC**	**< 62 mg/dL (< 0.5‰)**	**62–< 136 mg/dL (0.5–< 1.1‰)**	**136–< 247 mg/dL (1.1–< 2.0‰)**	**≥ 247 mg/dL (≥ 2.0‰)**	**p ***
n	1,093	187	60	108	76	
Severe TBI, %	29.5	33.2	43.3	46.3	36.8	< 0.001
ICU treatment, %	86.3	89.3	86.7	92.6	90.8	0.043
Mechanical ventilation, %	46.4	46.6	45.5	59.2	52.7	0.035
In-hospital mortality, %	12.7	10.7	11.7	7.4	10.5	0.139
Mortality, aOR	Reference	0.92 (0.47–1.79)	1.02 (0.33–3.14)	0.68 (0.28–1.67)	1.16 (0.45–3.01)	0.922 †
ICU admission, aOR	Reference	1.34 (0.80–2.22)	1.02 (0.45–2.32)	1.77 (0.84–3.75)	1.51 (0.68–3.39)	0.413 †

* Linear-by-linear association (trend); † global test of the category variable. Length of ICU stay and duration of ventilation showed no trend across categories (Jonckheere–Terpstra test p = 0.751 and p = 0.831).

Supplementary Figure S1: Flow diagram of patient selection. ALC, alcohol-positive (serum ethanol > 1 mg/dL, laboratory detection limit); noALC, alcohol-negative.

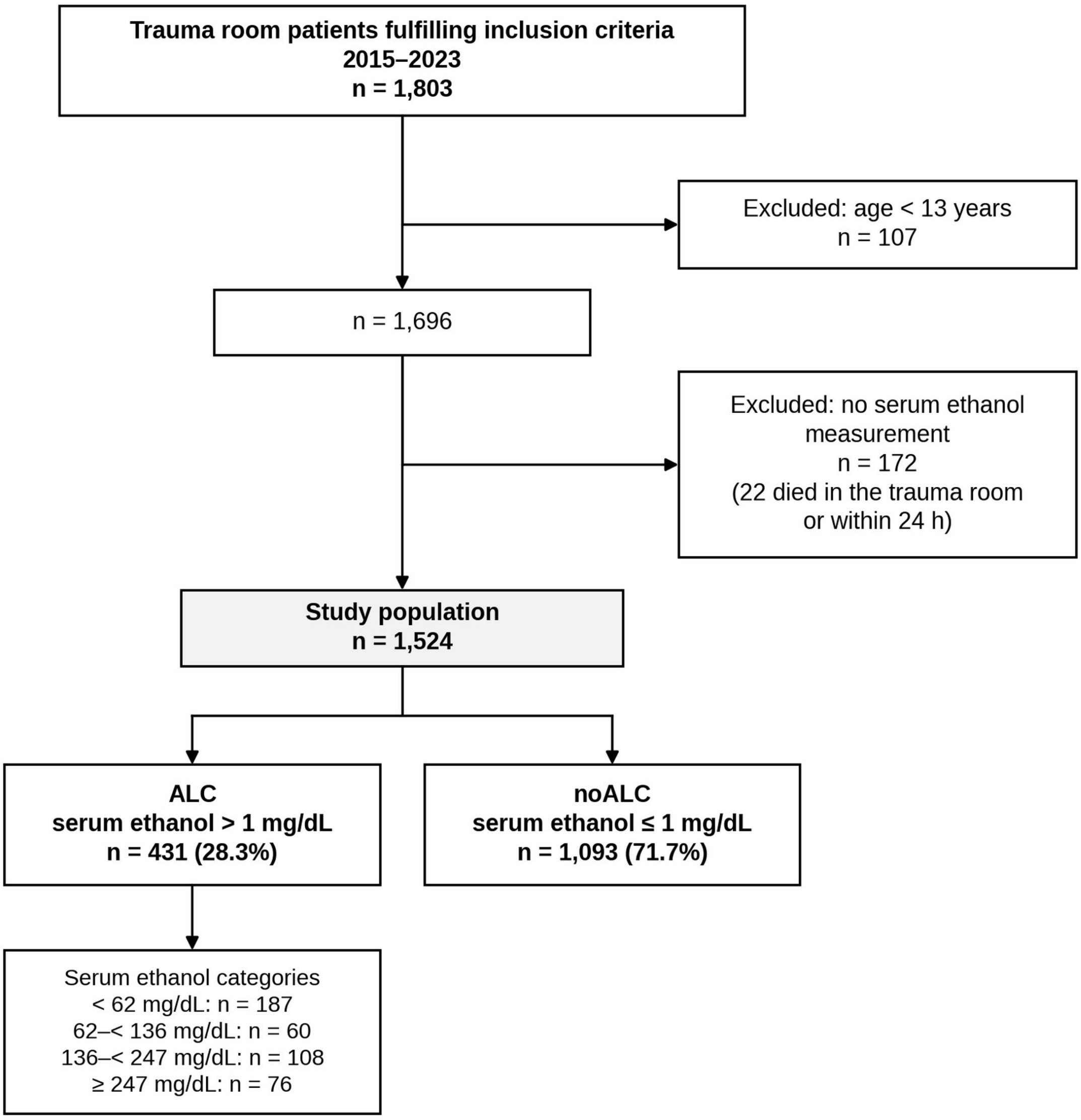

Supplement: Supplementary file 1 [file 12245_2026_1411_MOESM1_ESM.pdf]
